# Supplementary material for: YAP1 nuclear efflux and transcriptional reprograming follow membrane diminution upon VSV-G-induced cell fusion
Source: Nat Commun. 2021 Jul 23;12:4502. doi: 10.1038/s41467-021-24708-2 (PMC8302681; doi:10.1038/s41467-021-24708-2)
Supplement: Supplementary file 1 — Supplementary Information [file 41467_2021_24708_MOESM1_ESM.pdf]

YAP1 nuclear efflux and transcriptional reprograming follow membrane diminution upon VSV-G-induced cell fusion

Daniel Feliciano*[1], Carolyn M. Ott[1], Isabel Espinosa-Medina[1], Aubrey V. Weigel[1], Lorena Benedetti[1], Kristin M. Milano[2], Zhonghua Tang[2], Tzumin Lee[1], Harvey J. Kliman[2], Seth M. Guller[2], and Jennifer Lippincott-Schwartz*[1]

[1]Janelia Research Campus, Howard Hughes Medical Institute, Ashburn, VA, 20147, USA
[2]Department of Obstetrics, Gynecology and Reproductive Sciences, Yale University School of Medicine, New Haven, CT, 06520, USA

*Corresponding author: lippincottschwartzj@janelia.hhmi.org
*Corresponding author: felicianod@janelia.hhmi.org

Supporting Information contains:

- **Supplementary Figures**
- **Supplementary Table 1**

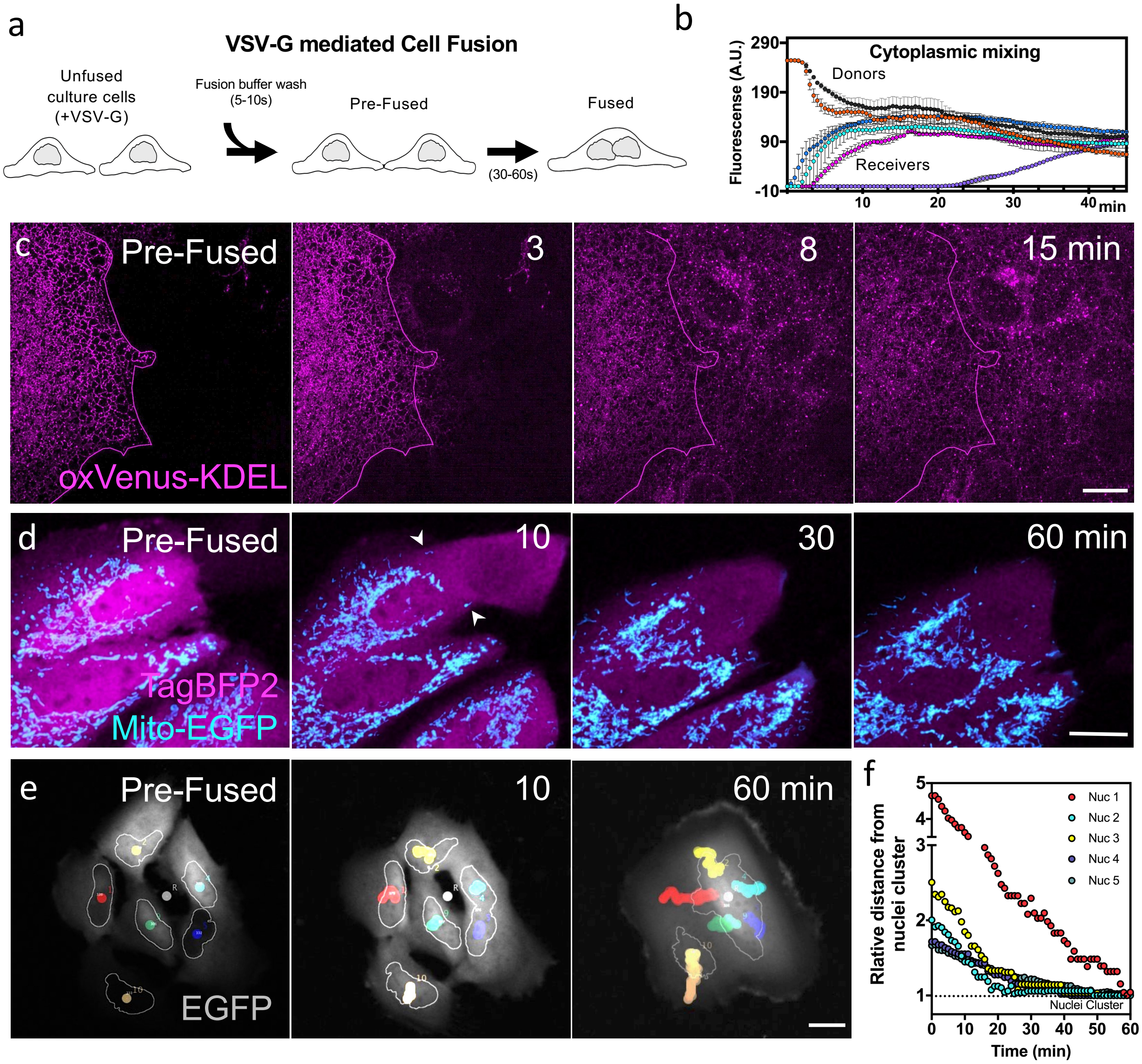
a
VSV-G mediated Cell Fusion
Unfused culture cells (+VSV-G)
Fusion buffer wash (5-10s)
Pre-Fused
(30-60s)
Fused
b
Cytoplasmic mixing
Donors
Receivers
Fluorescense (A.U.)
290
190
90
-10
10
20
30
40 min
c
Pre-Fused
3
8
15 min
oxVenus-KDEL
d
Pre-Fused
10
30
60 min
TagBFP2
Mito-EGFP
e
Pre-Fused
10
60 min
EGFP
f
Rlative distance from nuclei cluster
Nuc 1
Nuc 2
Nuc 3
Nuc 4
Nuc 5
Nuclei Cluster
Time (min)
0
10
20
30
40
50
60
1
2
3
4
5

Supplementary Figure 1. **Intermixing of small cytoplasmic proteins and organelles during cell fusion.** (a) Diagram of VSV-G mediated cell fusion. Cells expressing VSV-G and different subcellular markers are rapidly washed (5-10 seconds) with Fusion Buffer to induce cell fusion. Fusion of cells occurs 30-60 seconds after wash. (b) Exchange of a small fluorescent cytoplasmic protein (mEmerald) between Donor cells (orange and black curves) and Receiver cells during cell fusion (observed as a measured decrease in fluorescence intensity in Donors and an increase in fluorescence intensity in Receivers). Note that, depending on the time of fusion, the rate of cytoplasmic mixing varies. Each curve is the average fluorescence of the cytoplasmic marker at 3 different ROIs within each cell. (c) Cells expressing either VSV-G or VSV-G and an ER marker (oxVenus-KDEL) were cultured together and then fused. ER mixing was monitored as the ER marker spread into Receiver cells. (d) Cells expressing only VSV-G or VSV-G, a cytoplasmic marker (TagBFP2), and a mitochondria marker (Mito-EGFP) were cultured together and then fused. Initial fusion is observed when the cytoplasmic marker enters the Receiver cells, and mitochondria mixing is determined by the gain of the labeled mitochondria. (e and f) To assess nuclear clustering, cells transiently co-transfected with VSV-G and the nuclear marker H2B-mCherry were imaged live during and after cell fusion, then nuclei displacement and clustering were tracked (e). The movement of multiple nuclei simultaneously within the same syncytium is displayed relative to their individual distances from the nuclei cluster at different time points after cell fusion. Different tracks (nuclei) are depicted in different colors (See also Supplementary Video 5). Images in c, d, and e are representative of 4 independent experiments. All imaging in this figure is confocal microscopy. Scale bars = 10 µm.

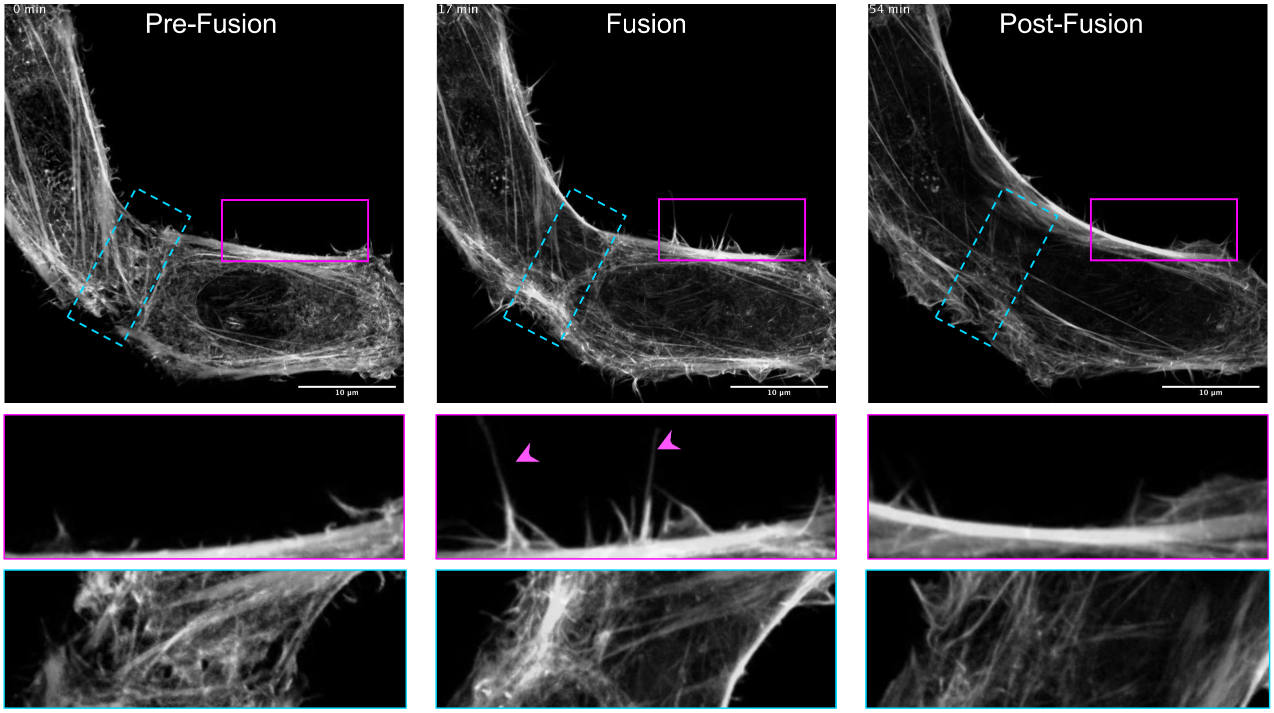

Supplementary Figure 2. **Remodeling of the actin cytoskeleton after VSV-G induced cell fusion.** Stable U2OS cells expressing lifeact-EGFP (F-actin) and tranfected with VSV-G were imaged using AiryScan microscopy (Zeiss). Differences in actin structures were observed between Pre-Fusion (15 seconds after Fusion Buffer wash), Fusion (17 min after Fusion Buffer wash) and Post-Fusion (54 min after Fusion Buffer wash) states. Cyan dotted squares highlight the organization of the actin cytoskeleton within the fusion interface at different time points (lower insets). Higher magnification insets (top insets) of magenta ROIs shows changes in actin structures. Magenta arrow heads point to the filopodia formed upon cell fusion. Images are representative of 3 independent experiments. (See also Supplementary Video 4) Scale bars = 10 μm.

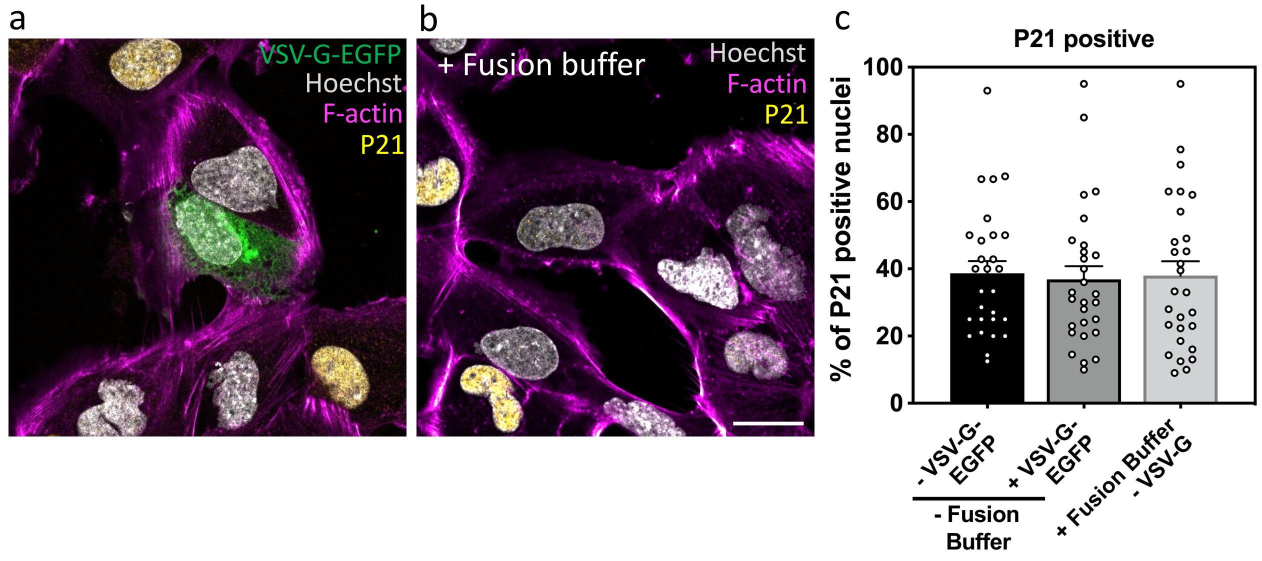

Supplementary Figure 3. **VSV-G transfection or incubation with fusion buffer does not promote increase in P21 positive nuclei in non-fused cells.** (a) Representative images of immunofluorescence staining of P21 positive nuclei in non transfected or VSV-G-EGFP transfected SUM-159 cells. These cells were not treated with Fusion Buffer. (b) Additionaly, non tranfected SUM-159 cells treated with Fusion Buffer were stained for P21. (a,b) All cells were imaged by confocal microscopy (c) The percentage of P21 positive nuclei was quantified. Error bars represent the SEM, n=28, n=28, and n=28 cells examined over 3 independent experiments for all conditions. Scale bars = 10 µm.

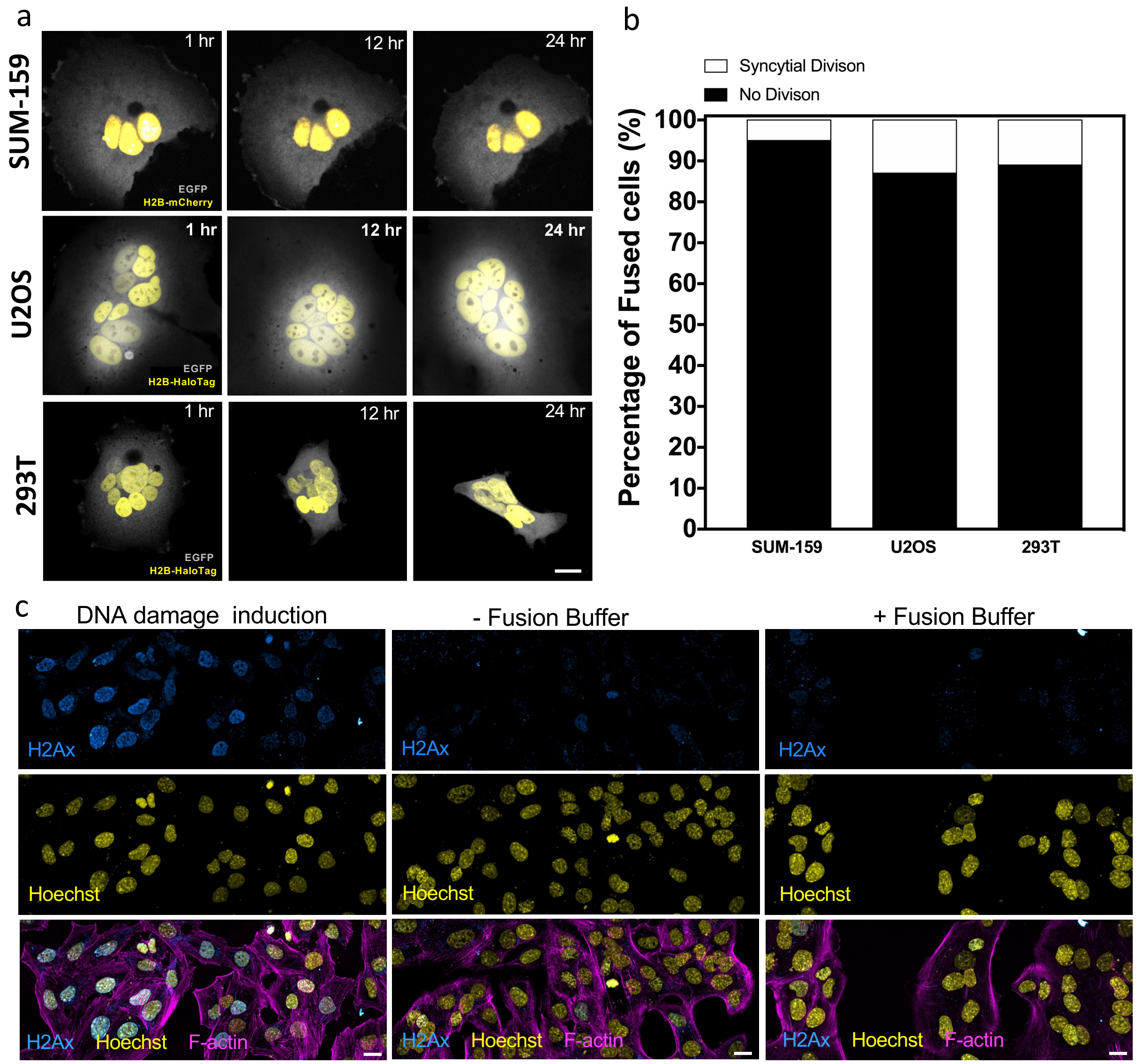

Supplementary Figure 4. **Cell division stops after VSV-G mediated cell fusion independent of DNA damage.** (a) Cell division was quantified by live cell imaging of SUM-159 cell expressing cytoplasmic (EGFP) and nuclear (H2B-mCherry or H2B-Halo Tag) markers. Representative confocal images of fused SUM-159 (n=20), U2OS (n=25), and HEK 293T (n=40) cells monitored for 24 hr after VSV-G mediated fusion are shown. (b) 88-95% of fused cells no longer divided after cell fusion. (c) Assessment of DNA damage after cell fusion. SUM-159 cells were stained with antibodies against the DNA damage marker H2Ax before and after induction of cell fusion. Cells induced for DNA damage and stained for H2Ax were used as positive control for DNA damage. Images are representative of 3 independent experiments. Scale bars = 10 µm.

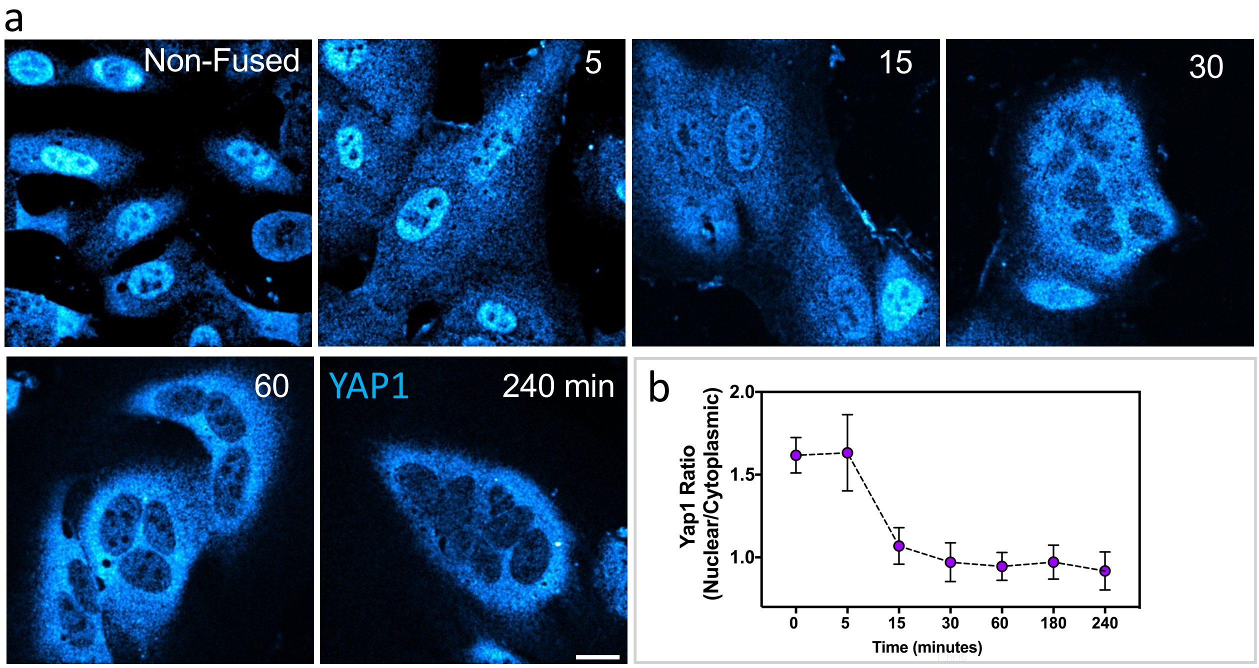

Supplementary Figure 5. **YAP1 cytoplasmic localization is observed in SUM-159 fused cells.** (a) Unwashed or Fused SUM-159 cells were fixed and immunostained using antibodies against YAP1 at different times during and after fusion, then imaged by confocal microscopy. (b) The YAP1 nuclear to cytoplasmic ratios were measured. Error bars represent the SEM, n=28, n=15, n=16, n=16, n=16, n=15 and n=16 cells examined over 3 independent experiments for 0, 5, 15, 30, 60, 180, and 240 min, respectively. Scale bars = 10 μm.

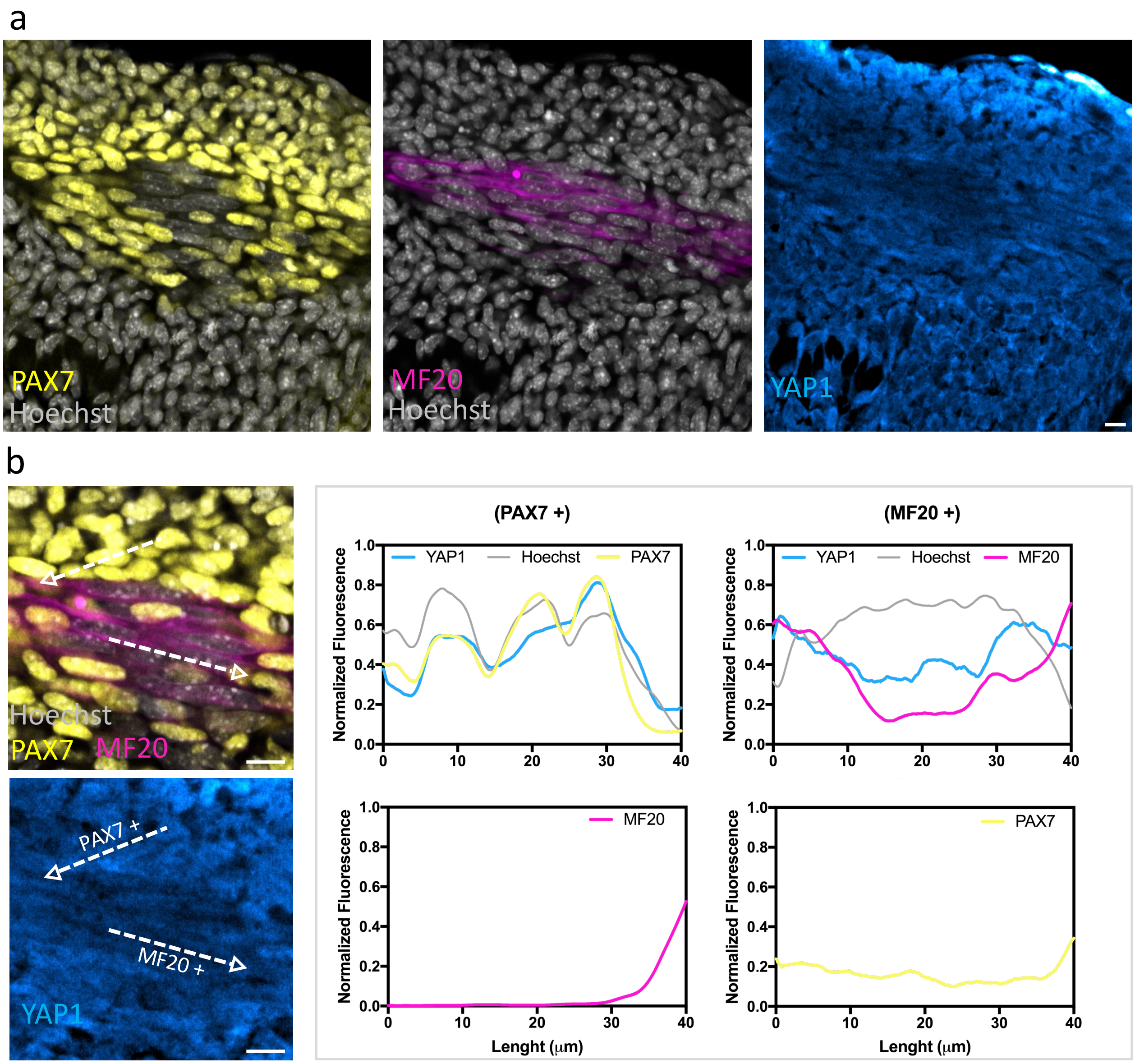

Supplementary Figure 6. **YAP1 inhibition during mouse skeletal muscle development.** (a) 25 μm sections of mouse E10.5 skeletal muscle were fixed, immunostained with anti-YAP1, anti-PAX7 and anti-MF20 antibodies and imaged by confocal microscopy to determine YAP1 localization. Progenitor muscle cells (non-fused) labeled with PAX7 (localized in the nucleus) while fused muscle cells label with MF20 antibodies. Images are representative of 3 independent experiments (from 2 E10.5 embryos). (b) Line scan (dotted line arrow) analyses were performed to compare YAP1 distribution between PAX7+ and MF20 + muscle cells. The normalized fluorescence of PAX7, MF20, and the nuclear marker Hoechst were also included for reference. Scale bar size = 10 μm.

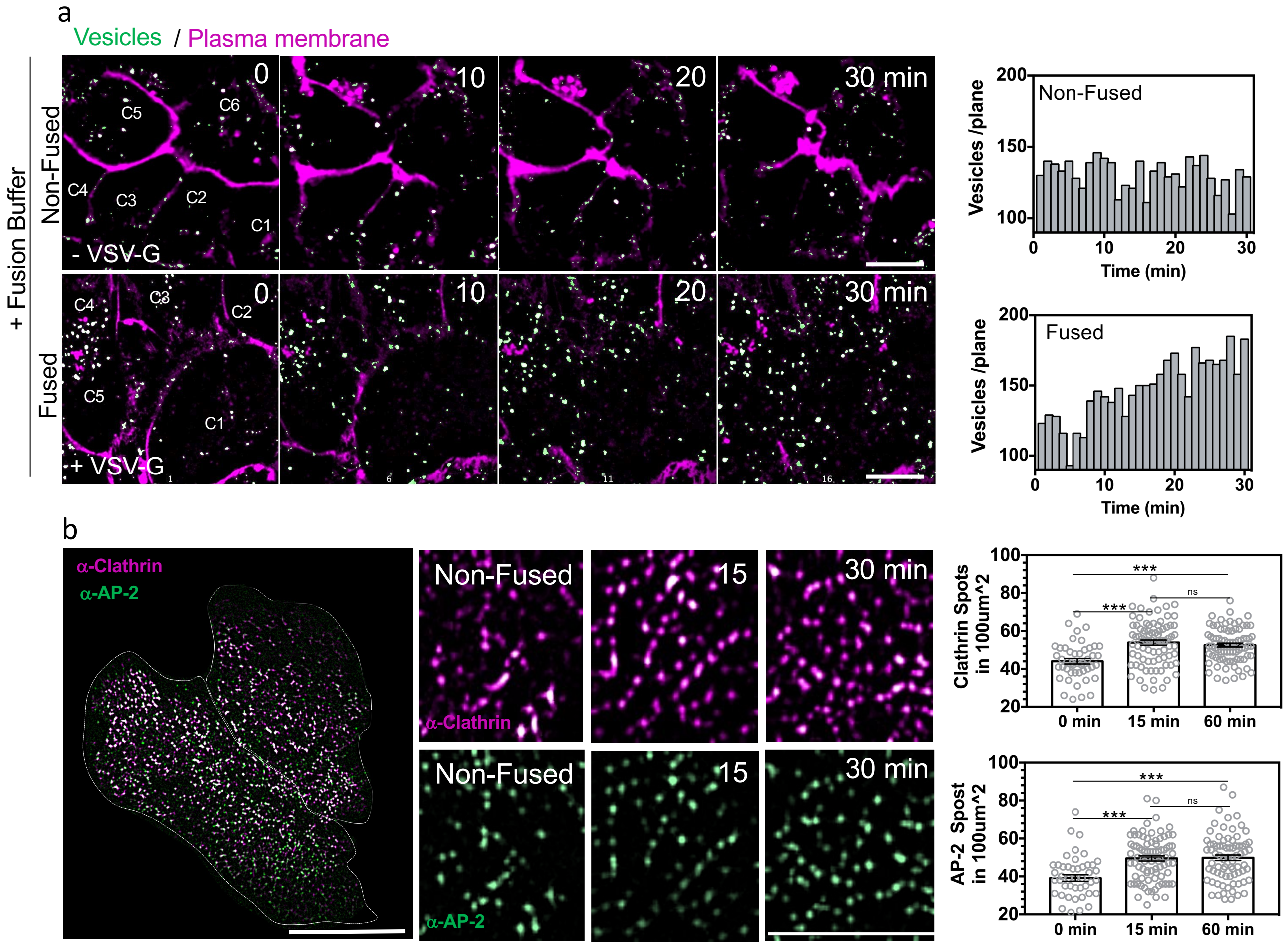

Supplementary Figure 7. **Clathrin-mediated endocytosis increases after cell fusion.** (a) Non transfected or VSV-G transfected SUM-159 cells were stained with a lipid fluorescent dye (DiD) to monitor internalizing vesicles by confocal microscopy upon fusion buffer wash (PM in magenta, mask detecting internalized vesicles in green). Images are representative of 3 independent experiments. (b) Immunofluorescence TIRF-microscopy of SUM-159 cells double labeled with antibodies to the clathrin heavy chain (CHC) and the alpha-subunit of the clathrin adaptor protein 2 (AP-2). The number of positive endocytic sites (spots) per 100 μm^2 were quantified before (Non-Fused) and after fusion (15, 30 min). For both CHC and AP-2 the error bars represent the SEM, n=46, n=81, n=78 images (100 μm^2) examined over 3 independent experiments for 0, 15, and 60 min, respectively. $P < 0.0001$ (***). Not significant = ns ($P > 0.05$). Statistical significance calculations were performed using a two-tailed unpaired Student's *t*-test. Scale bar size = 10μm

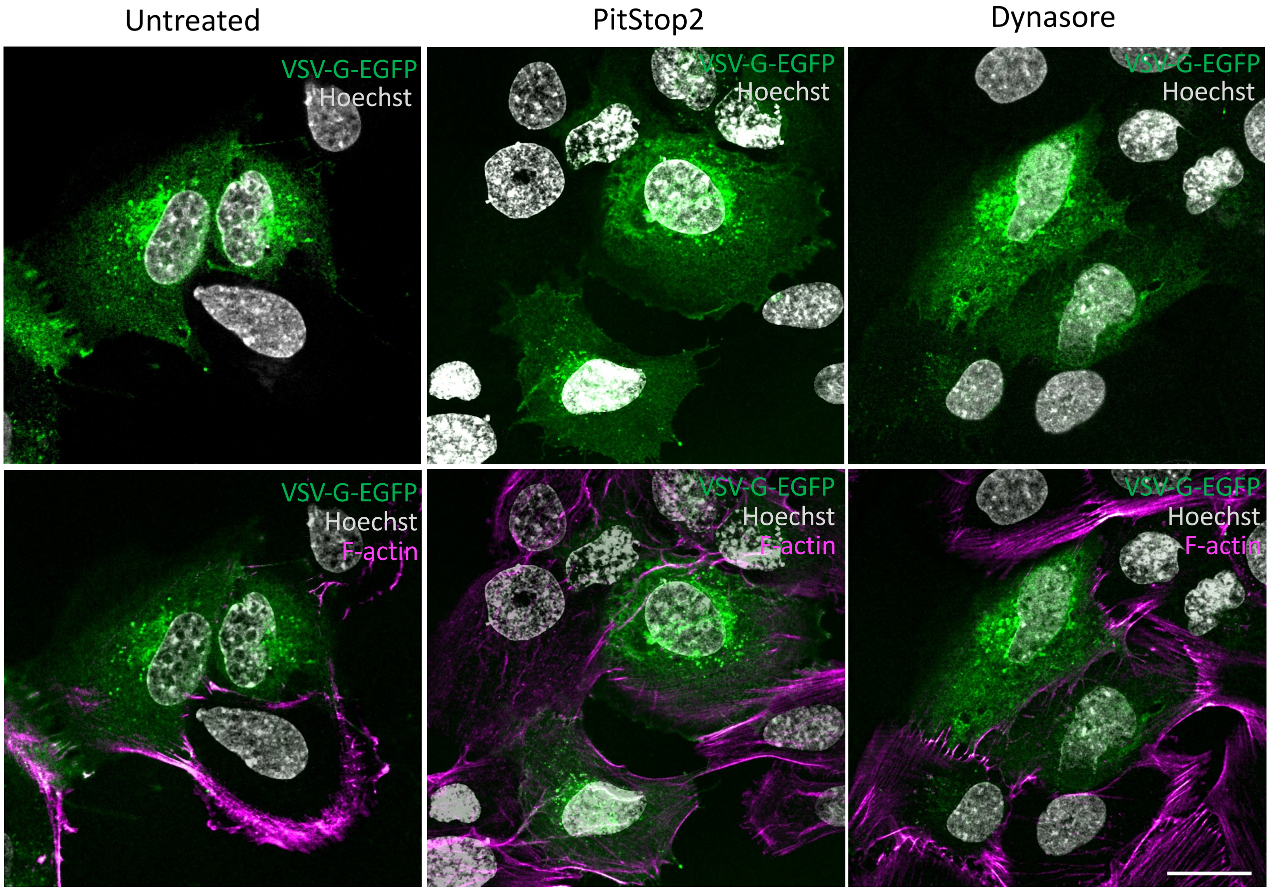

Supplementary Figure 8. **Inhibitors of endocytosis do not perturb VSV-G cellular distribution.** SUM-159 cells were transfected with VSV-G-EGFP. Cells were fixed, stained with Hoechst and phalloidin, and imaged by confocal microscopy. VSV-G-EGFP distribution is not altered by treatment with PitStop2 or Dynasore. Images are representative of 5 independent experiments. Cells were not washed with fusion buffer. Scale bar size = 10 μm

a

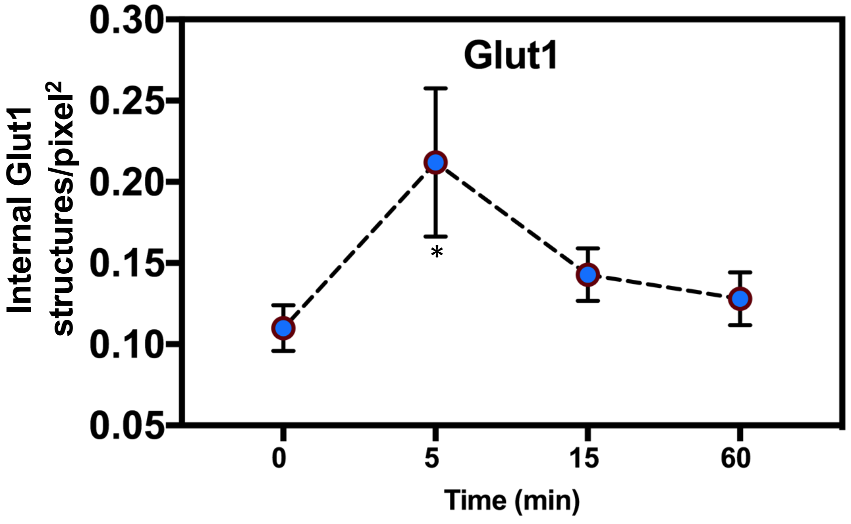
Glut1
Internal Glut1 structures/pixel^2
0.30
0.25
0.20
0.15
0.10
0.05
*
0
5
15
60
Time (min)

b

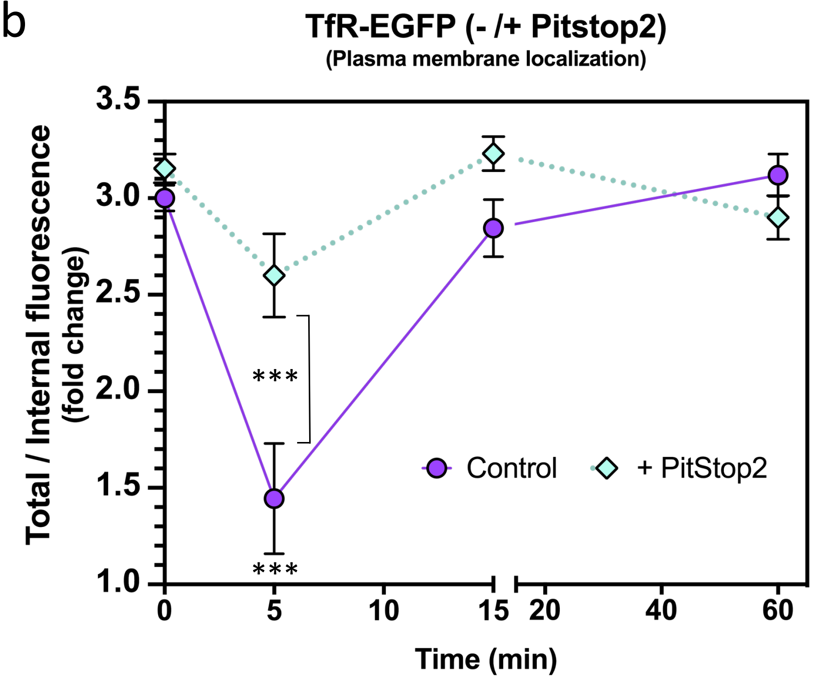
TfR-EGFP (- /+ Pitstop2)
(Plasma membrane localization)
Total / Internal fluorescence (fold change)
3.5
3.0
2.5
2.0
1.5
1.0
***
***
Control
+ PitStop2
0
5
10
15
20
40
60
Time (min)

Supplementary Figure 9. **Internalization of endocytic cargo upon cell fusion.** (a) SUM-159 cells expressing VSV-G were induced to fuse and then fixed at the indicated time points, stained with anti-Glut1 antibodies and imaged by confocal microscopy. Internal Glut1 structures were quantified at different time points before or after fusion (0, 5,15, 60 min). Error bars represent the SEM, n=15, n=10, n=8, and n=10 cells for 0, 5, 15, 30, and 60 min, respectively. P=0.0198 (*) (b) HEK 293T cells transfected with both VSV-G and the transferrin receptor (TfR-EGFP) were cultured with or without PitStop2, and then induced to fuse. Internalization of TfR-EGFP was quantified using a bromophenol blue (BPB) quench-assisted localization assay. The fluorescence intensity before and after surface quenching with BPB were quantified at different time points before or after fusion (0, 5,15, 60 min). The ratio of Total to Internal fluorescence is displayed (see Methods). Error bars represent the SEM. For untreated (control, magenta line) cells: n=29, n=27, n=22, and n=26 cells at 0, 5, 15, and 60 min, respectively. For PitStop2 treated cells (cyan dotted line): n=21, n=19, n=12, and n=17 cells at 0, 5, 15, and 60 min, respectively. Statistical differences are between non-fused (0 min) and fused (5 min, P<0.0001 ***) cells or between untreated and PitStop treated fused (5 min, P<0.0001 ***) cells. Statistical significance calculations were performed using a two-tailed unpaired Student's *t*-test.

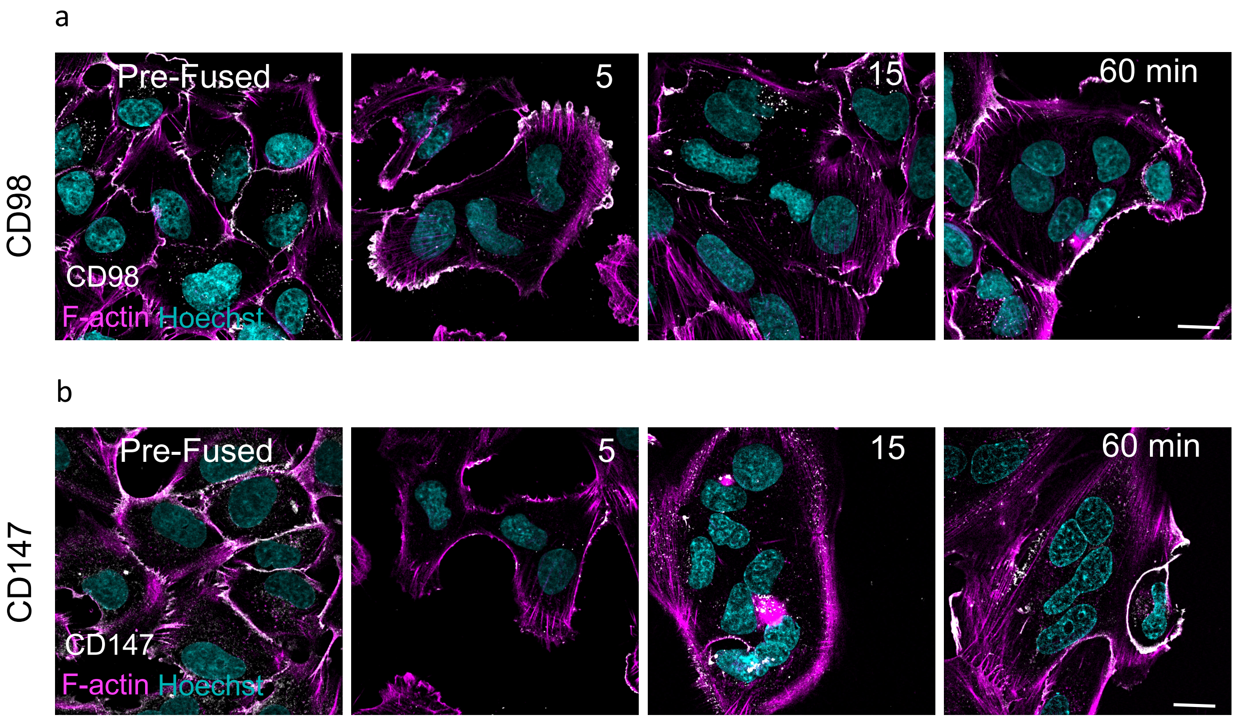

Supplementary Figure 10. **Clathrin-independent endocytic cargo does not internalize upon cell fusion.** SUM-159 cells expressing VSV-G were induced to fused and then were fixed at different time points. Anti-CD98 (a) and Anti-CD147 (b) antibodies were used to assess the subcellular localization of these clathrin-independent endocytic cargos during cell fusion using confocal microscopy. No detectable internalization of these cargos was observed. Images in a and b are representative of 3 independent experiments. Scale bars = 10 μm.

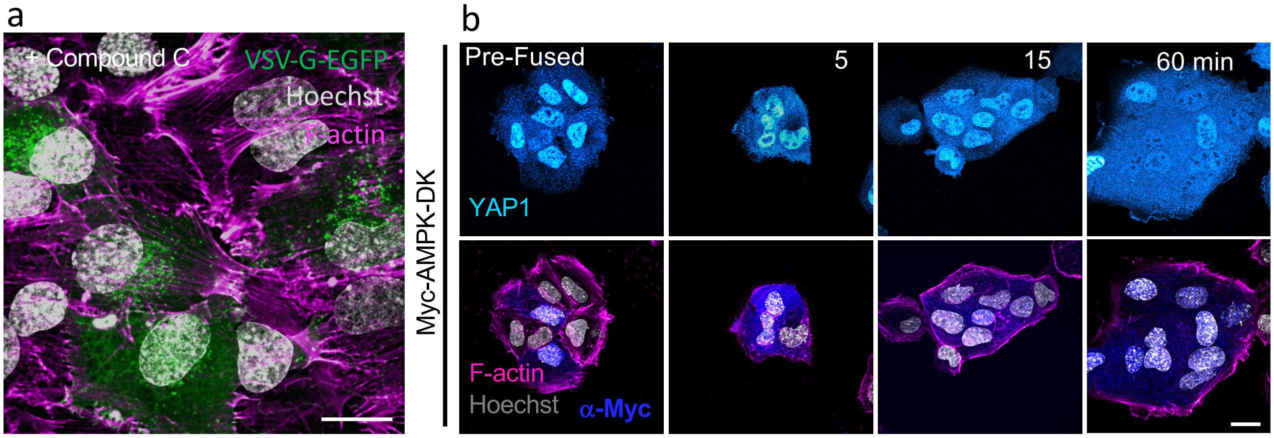

Supplementary Figure 11**. AMPK inhibition does not disrupt VSV-G localization, but blocks YAP1 nuclear eviction**. (a) SUM-159 cells were transfected with VSV-G-EGFP. Cells were fixed and imaged by confocal microscopy to asses the effect of compound C treatment on VSV-G-EGFP cellular localization. Cells were not washed with Fusion Buffer. (b) SUM-159 cells transfected with the Myc-tagged dominant negative form of the α2 subunit of AMPK (Myc-AMPK-DK), were induced to fuse and then fixed at indicated time points and immunostained with anti-YAP1 and anti-Myc antibodies to detect YAP1 localization withing Myc-AMPK-DK transfected cells. Images in a and b are representative of 3 and 5 independent experiments, respectively. Scale bars = 10 µm.

Suppementary Table 1. Reagents used for quantitative real-time PCR (see Methods section)

Reagent	Supplier	Cat#
TaqMan RNA-to-Ct 1 step kit	ThermoFisher	432938

Gene Expression Assays	Supplier	Cat#	Assay ID #
CDKN1A	ThermoFisher	4331182	Hs00355782_m1
GAPDH	ThermoFisher	4331182	Hs02786624_g1
PGK	ThermoFisher	4331182	Hs00943178_g1
