# Supplementary material for: YAP1 nuclear efflux and transcriptional reprograming follow membrane diminution upon VSV-G-induced cell fusion
Source: Nat Commun. 2021 Jul 23;12:4502. doi: 10.1038/s41467-021-24708-2 (PMC8302681; doi:10.1038/s41467-021-24708-2)
Supplement: Supplementary file 3 — Description of Additional Supplementary Files [file 41467_2021_24708_MOESM3_ESM.pdf]

Description of Additional Supplementary Files

File Name: Supplementary Movie 1
Description: Cytoplasmic mixing after cell fusion of SUM-159 cells. Cells 1255 expressing VSV-G alone (Receiver cells) or co-expressing VSV-G and a cytoplasmic 1256 marker (Donor cells, cytoplasmic mEmerald in cyan) were mixed and fused by a brief 1257 wash with Fusion Buffer and then were imaged by confocal microscopy at an acquisition 1258 rate of 1 frame every minute for 1hr. Nuclei are labeled by Hoechst (yellow). Related to 1259 Figure 1a and b.

File Name: Supplementary Movie 2
Description: Lattice light sheet microscopy of fusion pore formation and 1262 plasma membrane remodeling after cell fusion. Cells expressing VSV-G and a plasma 1263 membrane marker (mEmerald-GPI, in cyan) were induced to fuse and changes in the 1264 plasma membrane were assessed by lattice-light sheet microscopy at an acquisition 1265 rate of 1 volume every 30 seconds for 33 minutes. Related to Figure 1c.

File Name: Supplementary Movie 3
Description: Lattice light sheet microscopy of fusion pore formation and 1268 plasma membrane remodeling after cell fusion (orthogonal view) (mEmerald-GPI). 1269 Related to Figure 1c (lower panel) and d.

File Name: Supplementary Movie 4
Description: Remodeling of the actin cytoskeleton during cell fusion. U2OS 1272 cells stably expressing lifeact-EGFP (F-actin) and tranfected with VSV-G were imaged 1273 and the dynamics of filamentous actin were monitored after washing with Fusion Buffer. 1274 Images were acquired using AiryScan microscopy (Zeiss) at an acquisition rate of 1 1275 frame every minute for 1hr. Color coding represent the Z-position of actin filaments. 1276 Scale bars = 10µm. Related to Supplementary Figure 2.

File Name: Supplementary Movie 5
Description: Cell fusion promotes nuclei clustering. To assess nuclear 1279 clustering, cells transiently co-transfected with VSV-G, cytoplasmic EGFP (gray), and 1280 the nuclear marker H2B-mCherry (blue) were imaged live by confocal microscopy 1281 during cell fusion at an acquisition rate of 1 frame every minute for 1hr, then nuclei 1282 displacement and clustering were tracked. Different tracks (nuclei) are depicted in 1283 different colors. Related to Supplementary Figure 1.

File Name: Supplementary Movie 6
Description: Cell fusion remodeling of the plasma membrane reduces total 1286 cellular surface area. SUM-159 cells expressing VSV-G and a PM marker (CAAX1287 EGFP) were imaged by confocal microscopy at an acquisition rate of 1 frame every 1288 minute for 40 minutes as cells fused (Z-stacks were used to generate three-dimensional 30 1289 models of cells). The surface of fusing cells is colored based on the surface area as 1290 measured by the IMARIS surface tool. Related to Figure 4.

File Name: Supplementary Movie 7
Description: The number of endogenous AP-2- EGFP positive endocytic 1293 sites increases in fusing cells but not in non-fusing cells. CRISPR-Cas9 gene edited 1294 SUM-159 cells expressing

endogenous AP-2 –EGFP and transfected with VSV-G, were induce to fused, and the density of AP-2 –EGFP at the plasma membrane (grey puncta) was measured overtime by TIRF microscopy at an acquisition rate of 1 frame every 16.5 seconds for 44 minutes.To determine T=0 in fusing cells, cytoplasmic mixing was monitored (TagBFP2, blue). Related to Figure 4.
