# Supplementary material for: YAP1 nuclear efflux and transcriptional reprograming follow membrane diminution upon VSV-G-induced cell fusion
Source: Nat Commun. 2021 Jul 23;12:4502. doi: 10.1038/s41467-021-24708-2 (PMC8302681; doi:10.1038/s41467-021-24708-2)
Supplement: Supplementary file 11 — Reporting Summary [file 41467_2021_24708_MOESM11_ESM.pdf]

nature research

Corresponding author(s): Jennifer Lippincott-Schwartz

Last updated by author(s): Jun 1, 2021

Reporting Summary

Nature Research wishes to improve the reproducibility of the work that we publish. This form provides structure for consistency and transparency in reporting. For further information on Nature Research policies, see our Editorial Policies and the Editorial Policy Checklist.

Statistics

For all statistical analyses, confirm that the following items are present in the figure legend, table legend, main text, or Methods section.

n/a	Confirmed	
☐	☒	The exact sample size (n) for each experimental group/condition, given as a discrete number and unit of measurement
☐	☒	A statement on whether measurements were taken from distinct samples or whether the same sample was measured repeatedly
☐	☒	The statistical test(s) used AND whether they are one- or two-sided<br>*Only common tests should be described solely by name; describe more complex techniques in the Methods section.*
☒	☐	A description of all covariates tested
☒	☐	A description of any assumptions or corrections, such as tests of normality and adjustment for multiple comparisons
☐	☒	A full description of the statistical parameters including central tendency (e.g. means) or other basic estimates (e.g. regression coefficient) AND variation (e.g. standard deviation) or associated estimates of uncertainty (e.g. confidence intervals)
☐	☒	For null hypothesis testing, the test statistic (e.g. F, t, r) with confidence intervals, effect sizes, degrees of freedom and P value noted<br>*Give P values as exact values whenever suitable.*
☒	☐	For Bayesian analysis, information on the choice of priors and Markov chain Monte Carlo settings
☒	☐	For hierarchical and complex designs, identification of the appropriate level for tests and full reporting of outcomes
☒	☐	Estimates of effect sizes (e.g. Cohen's d, Pearson's r), indicating how they were calculated

Our web collection on statistics for biologists contains articles on many of the points above.

Software and code

Policy information about availability of computer code

Data collection	We used Zen black edition v2.1 software from Zeiss to collect confocal and airyscan images. In addition we use d NIS-Elements Ar v4.30 from Nikon to collect confocal and TIRF images.
Data analysis	We used Imaris v8.1 (Bitplane) to reconstruct three-dimensional images and measure surface areas and volumes. We used ImageJ v2.1.0 to perform fluorescent intensities measurements, perform nuclear tracking , Yap1 nuclear/cytoplasm ratio calculations, and image visualization. We used GraphPad Prism8 to perform statistical analysis.

For manuscripts utilizing custom algorithms or software that are central to the research but not yet described in published literature, software must be made available to editors and reviewers. We strongly encourage code deposition in a community repository (e.g. GitHub). See the Nature Research guidelines for submitting code & software for further information.

Data

Policy information about availability of data

All manuscripts must include a data availability statement. This statement should provide the following information, where applicable:

- Accession codes, unique identifiers, or web links for publicly available datasets
- A list of figures that have associated raw data
- A description of any restrictions on data availability

Data supporting the findings of this manuscript are available from the corresponding author upon reasonable request.
All RNA-seq data generated as a part of this study have been deposited in the National Center for Biotechnology Information Gene Expression Omnibus (GEO) and are accessible through the GEO Series accession number GSE168125.

Field-specific reporting

Please select the one below that is the best fit for your research. If you are not sure, read the appropriate sections before making your selection.

- [x] Life sciences
- [ ] Behavioural & social sciences
- [ ] Ecological, evolutionary & environmental sciences

For a reference copy of the document with all sections, see nature.com/documents/nr-reporting-summary-flat.pdf

Life sciences study design

All studies must disclose on these points even when the disclosure is negative.

Sample size	No statistical methods were used to pre-determine the sample sizes. At least 15 images were taken for each experiment involving immunofluorescence. The sample sizes were considered sufficient given that large differences, with a P value lower than 0.01, between the two experimental conditions were usually detected. All of the quantitative data shown represent the mean±S.E.M., exept when otherwise stated in the legend. Bar plots have been overlaid with dot plots showing all of the individual measured data points. No strongly scattering data points were excluded; all quantitative evaluation data points were taken into account and averaged to fully represent biological and technical variabilities.
Data exclusions	No data is excluded from this study.
Replication	At least 15 images are taken for each experiment, and in most cases 3 repeats are performed for each experiment.<br>All attempts at replication were successful.
Randomization	Some experiments were randomized and the investigators were blinded.
Blinding	The investigator is blinded to allocation during experiments and outcome assessment.

Reporting for specific materials, systems and methods

We require information from authors about some types of materials, experimental systems and methods used in many studies. Here, indicate whether each material, system or method listed is relevant to your study. If you are not sure if a list item applies to your research, read the appropriate section before selecting a response.

Materials & experimental systems

n/a	Involved in the study	
[ ]	[x]	Antibodies
[ ]	[x]	Eukaryotic cell lines
[x]	[ ]	Palaeontology and archaeology
[ ]	[x]	Animals and other organisms
[ ]	[x]	Human research participants
[x]	[ ]	Clinical data
[x]	[ ]	Dual use research of concern

Methods

n/a	Involved in the study	
[x]	[ ]	ChIP-seq
[x]	[ ]	Flow cytometry
[x]	[ ]	MRI-based neuroimaging

Antibodies

Antibodies used	anti-p21 (Cell Signaling, Cat. # 2947S), anti-pH3 (anti- Phospho-Histone H3 (Ser10); Cell Signaling, Cat. #3377), anti-clathrin heavy chain (Abcam, Cat. # ab21679), anti-AP-2 (Abcam, Cat. # ab189995), anti-Glut1 (Abcam, Cat. # ab40084), anti-CD98 (BioLegend, Cat. # 315602), anti-CD147 (BioLegend, Cat. # 306202), anti-Yap1 (Cell Signaling #14074S), anti-phospho-YAP1 (Cell Signaling (S127) #13008), anti-AMPK (Cell Signaling, Cat. # 2532s), anti-phospho-AMPK (Cell Signaling, Cat. # 2531s), anti-Vinculin (Sigma, Cat. # V9131), anti-Tubulin (Sigma), anti-Pax7, Mouse IgG1, (DSHB, ID:AB528428), anti-MF20, Mouse IgG2b (DSHB, ID:AB2147781), anti-mouse IgG2b Cy3 (Jackson Immunoresearch Laboratories 115-165-207), anti-mouse IgG2b A647 (Jackson Immunoresearch Laboratories 115-605-207, and anti-rabbit IgG A488 (Jackson Immunoresearch Laboratories 111-545-144.
Validation	All antibodies used in this study have been validated previously in the literature. Validation information for each antibody is available from the source vendors where the antibodies were purchased.

Eukaryotic cell lines

Policy information about cell lines

Cell line source(s)	The human mesenchymal triple-negative breast cancer-stem cell line SUM-159 was originally derived by Dr. Doug Schwartzentruber of the Surgery Branch (NCI). SUM-159-AP2-EGFP was generated in Dr. Tomas Kirchhausen lab at the

	Department of Cell Biology, Harvard Medical School. Both SUM-159 and SUM-159-AP2-EGFP were obtained as a gift from Tomas Kirchhausen. The C2C12, U2OS and HEK 293T cell lines were obtained directly from ATCC (CRL-1772, HTB96 and CRL11268, respectively.
Authentication	None of the cell lines are authenticated.
Mycoplasma contamination	We did not detect mycoplasma contamination in are cultures. Mycoplasma contamination is tested with MycoAlertTM Mycoplasma Detection Kit-Lonza.
Commonly misidentified lines (See ICLAC register)	No ICLAC cell lines have been used in this study.

Animals and other organisms

Policy information about studies involving animals; ARRIVE guidelines recommended for reporting animal research

Laboratory animals	10 weeks old pregnant CD-1 female mice were obtained from Charles River and the copulatory plug was labeled as day 0.5 dpc. At 10.5 dpc the mice were sacrificed by cervical dislocation and embryos were fixed.
Wild animals	No wild animals were used in the study.
Field-collected samples	No field collected samples were used in the study.
Ethics oversight	Procedures and protocols (17-152) on mice were approved by the Institutional Animal Care and Use Committee at Janelia Research Campus, Howard Hughes Medical Institute.

Note that full information on the approval of the study protocol must also be provided in the manuscript.

Human research participants

Policy information about studies involving human research participants

Population characteristics	Placentas from uncomplicated term pregnancies were collected within 30 min following elective cesarean section without labor at New Haven Hospital. Infection was excluded on the basis of standard clinical criteria (absence of fever,uterine tenderness, maternal / fetal tachycardia, foul vaginal discharge). Written informed consent was obtained from all participants before enrollment.Gestational age was established based on menstrual date confirmed by sonographic examination before 20 wk gestation.
Recruitment	If the standard clinical criteria was met, written informed consent was obtained from all participants before enrollment.
Ethics oversight	Approval for this study was granted by the Yale University School of Medicine Human Investigation Committee through our collaborator Dr. Seth Guller.

Note that full information on the approval of the study protocol must also be provided in the manuscript.
